# Characterization of the Protective Efficacy Against QX Strain of a Recombinant Infectious Bronchitis Virus With H120 Backbone and QX Spike Gene

**DOI:** 10.3389/fmicb.2022.883642

**Published:** 2022-06-17

**Authors:** Wenlian Weng, Qingyan Liu, Wenxiang Xue, Huan Wang, Shouguo Fang, Yingjie Sun, Lei Tan, Cuiping Song, Xusheng Qiu, Weiwei Liu, Chan Ding, Ying Liao

**Affiliations:** ^1^Department of Avian Diseases, Shanghai Veterinary Research Institute, Chinese Academy of Agricultural Sciences, Shanghai, China; ^2^College of Agriculture, College of Animal Sciences, Yangtze University, Jingzhou, China; ^3^Jiangsu Co-innovation Center for Prevention and Control of Important Animal Infectious Diseases and Zoonoses, Yangzhou University, Yangzhou, China

**Keywords:** infectious bronchitis virus, vaccine H120, QX, S gene, reverse genetics, rH120-QX(S)

## Abstract

Infectious bronchitis virus (IBV) has been prevalent in chicken farms for many years, and its control relies on extensive vaccine administration. The continuous emergence of new variants and the low cross-protection efficiency prompt the development of new vaccines. In this study, we develop a reverse genetics technique based on the classical vaccine strain H120 genome, *via in vitro* ligation method. Using the H120 genome as the backbone, we constructed the recombinant virus rH120-QX(S) by replacing the H120 S gene with the QX S gene, a prevalent strain in China. Biological characteristics of the rH120-QX(S) virus, such as 50% egg lethal dose (ELD_50_), 50% egg infectious dose (EID_50_), dwarf embryo, growth curve, and genetic stability, are measured, which are comparable to the parental virus H120. There are no clinical symptoms and tissue lesions in the trachea and kidney in the rH120-QX(S)-infected specific-pathogen-free (SPF) chickens, demonstrating that this recombinant virus does not confer pathogenicity. Furthermore, protection studies show that there is 100% homologous protection of rH120-QX(S) to the virulent QX strain, as shown by the absence of clinical signs and no lethality. Taken together, our results demonstrate that swapping the S gene onto the H120 genetic backbone is a precise and effective way to produce genetically defined IBV vaccine candidates.

## Introduction

Infectious bronchitis virus (IBV) belongs to *Nidovirale, Coronaviridae*, γ*-coronavirus* (Bande et al., 2017), which mainly infects chicken and causes an acute, highly contagious respiratory disease, imposing a great threat to the poultry industry. It is an enveloped virus, with a length of 27.6 kb positive-stranded genome. The gene 1 encodes polyprotein pp1a and pp1ab, the gene 2 encodes S protein, the gene 3 encodes 3a, 3b, and E protein, the gene 4 encodes M protein, the gene 5 encodes 5a and 5b, and the gene 6 encodes N protein (Faruku et al., 2015). The S protein is the largest structural protein with a size of ~128 kDa, which is a type I glycoprotein that protrudes the virion surface and is responsible for virus entry (Cavanagh, 2007; Jeanne et al., 2018). It is cleaved by the host protease into two subunits: S1 and S2. The S1 subunit is the head domain that contains the receptor binding domain (RBD) and harbors the main epitopes for neutralizing antibodies, responsible for the virus binding to cell surface receptors (Keep et al., 2020). The hypervariable region of the S1 subunit results in many serotypes. The S2 subunit is anchored into the viral membrane and is responsible for virus-cell membrane fusion (Edris et al., 2018).

Since the first report of IBV in 1931, many variants and serotypes/genotypes have been isolated (Wit et al., 2011; Li et al., 2019). The primary IBV serotypes found globally are QX, Mass-type, 4/91-like, D3128, D274-like, Italy 02, etc. These strains primarily cause respiratory symptoms, kidney lesions, and oviduct lesions (Bande et al., 2017). Long-term prevalence of IBV imposes serious challenges to the poultry industry by threatening sustainable poultry and egg supplies (Gallardo, 2021). The IBV genome replicates with a high rate of mutation, as the viral RNA-dependent RNA polymerase (RdRp) lacks proofreading capability (Zou et al., 2010). The frequent recombination and mutation result in the continual emergence of new strains and make the controlling of IB difficult and complex. Vaccination is the main strategy to control IBV in poultry farms. Live-attenuated vaccines are most commonly used in the farms, such as H120, H52, 4/91, Mac5 (Jordan, 2017). However, as these vaccines provide limited cross-protection, the chickens are often immunized with multiple serotype vaccines, which significantly increases the recombination risk among vaccines and field strains (Kottier et al., 1995). Thereby, vaccines with the same backbone and the main antigens of prevalent strain must be developed without the risk of generating a new strain by recombination.

The reverse genetics technique is a key tool for precisely and rapidly generating live-attenuated vaccine candidates (Bickerton et al., 2017). H120 is an attenuated live vaccine strain of the Massachusetts (Mass) serotype, which was obtained by passing the strain H into the chicken embryo up to the 120th passage, which was isolated in the Netherlands in 1956 (Bijlenga et al., 2004). In the recent 50 years, H120 was used worldwide as a primary vaccine in broilers, breeders, and layers. As IBV S protein harbors the main immune antigens, it is feasible to replace the H120 S gene with the corresponding S gene from pathogenic prevalent strains, to develop the recombinant vaccine candidates providing protection against homologous pathogenic prevalent strains (Armesto et al., 2011).

The IBV QX is one of the most popular genotypes across the world (Dolz et al., 2009; Cheng et al., 2017; Ganapathy et al., 2020). It is nephron-pathogenic and has become the predominant genotype in China since 1998 (Zhao et al., 2017). In this study, an infectious clone of vaccine strain H120 (rH120) was successfully generated by an *in vitro* ligation method. To develop the recombinant vaccine for the QX strain, a chimeric virus rH120-QX(S) was constructed by replacing the H120 S gene with the QX S gene, based on the H120 backbone. Compared with the field QX strain, the rH120-QX(S) strain is attenuated and provides 100% protection to the QX strain challenge. Thus, this recombinant virus is a promising vaccine candidate for the prevention of the predominant circulating genotype QX.

## Materials and Methods

### Virus and Cells

The H120 strain was purchased from Qingdao YEBIO Biological Engineering Co., Ltd., China. The QX strain, also referred to as IBV isolate SD (GenBank: KY421673.1), was a gift from Prof Zhang Guozhong's lab, China Agricultural University. The H120 and QX strains were propagated in the allantoic cavities of 10-day-old SPF chicken embryos. Embryos were inoculated with the virus and incubated for 36 h at 37°C before harvesting. BHK-21 cells were maintained in DMEM containing 10% FBS, 100 U/ml penicillin, and 100 μg/ml streptomycin.

### Embryos and Animals

Specific-pathogen-free chicken embryos were purchased from Jinan Sais Poultry CO. LTD (Shandong, China), which were hatched at 37.8°C with a relative humidity of 55–65% to produce 10-day-old SPF chicken embryos or to hatch 1-day-old chickens. All chickens were housed separately in isolators with consistent conditions, food and water were provided *ad libitum*.

### Construction of a Full-Length cDNA Clone of H120 and Generation of Recombinant Virus rH120-QX(S)

The viral RNA was extracted from 500 μl allantoic fluid infected with H120 or with QX, by using TRIzol reagent (Invitrogen, Carlsbad, CA), according to the manufacturer's manual. Reverse transcription was performed by M-MLV Reverse Transcriptase (Promega, USA) and Oligo (dT) primer. Using Q5^®^ High-Fidelity DNA Polymerases (New England Biolabs, USA), five cDNA fragments covering the H120 whole genome and the QX S gene were obtained from the cDNA template by PCR. The primers are based on the H120 sequence (GenBank accession number ON350836) and are listed in Supplementary Table 1.

**Figure 1:**
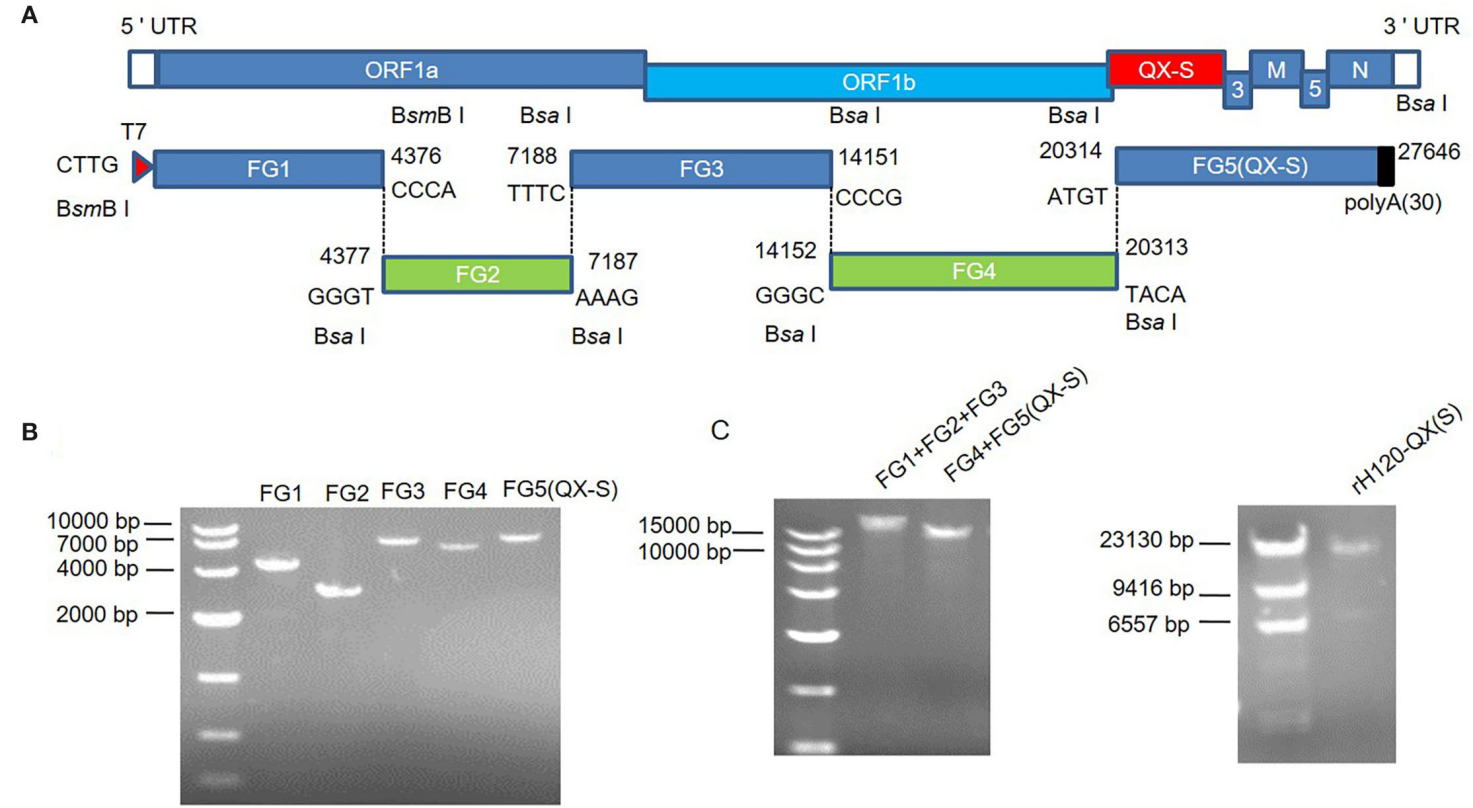
*In vitro* assembly of a full-length rH120-QX(S) infectious cDNA clone. **(A)** The schematic diagram for *in vitro* assembly of an infectious cDNA clone of rH120-QX(S). The genetic organization of rH120-QX(S) and the replacement of the H120 S gene with the QX S gene for the construction of rH120-QX(S) are shown. Five cDNA fragments covering the entire genome of the rH120-QX(S) strain were amplified by RT-PCR and cloned into the vector; the nucleotide sequence and location in the genome of the cohesive overhangs are indicated. A unique T7 promoter sequence was added at the 5'-terminus of FG1, and a poly-A (30 A) tail was added at the 3'-terminus of FG5. The terminal sequence of each cDNA fragment was franked with restriction enzyme sites B*sa* I or B*sm*B I. **(B)** The five fragments were cleaved from the plasmids and separated by agarose gel electrophoresis. **(C,D)** The intermediate ligation products [FG1+FG2+FG3 and FG4+FG5(QX-S)]. **(C)** and full-length cDNA of H120-QX(S) **(D)** were examined with agarose gel electrophoresis.

The cDNA fragments (FG2, FG3, FG4, and FG5) carry the restriction enzyme recognition sequence B*sa* I at both termini, and the cDNA fragment FG1 carries the restriction enzyme recognition sequence B*sm*B I at both termini, as shown in Figure 1. The five cDNA fragments were cloned into the pEASY^®^-Blunt-Zero vector (TransGen Biotech, China), respectively. To replace the H120 S gene with the QX S gene, the QX S and FG5(ΔS) (23803–27637 nt of the H120 genome) were fused by PCR and inserted into the pEASY^®^-Blunt-Zero vector. To facilitate the transcription of RNA from the cDNA, the T7 promoter sequence (TAATACGACTCACTATAGGG) was incorporated into the 5′-terminus of FG1. The five plasmids [pEASY^®^-Blunt-Zero-FG1, pEASY^®^-Blunt-Zero-FG2, pEASY^®^-Blunt-Zero-FG3, pEASY^®^-Blunt-Zero-FG4, and pEASY^®^-Blunt-Zero-FG5-QX (S)] cover the full-length cDNA of rH120 (ΔS) and QX (S).

Using enzymatic digestion from amplified plasmids, five cDNA fragments were obtained. The digestion products were separated by 1% agarose gel electrophoresis, and the bands corresponding to each fragment were cut from the gel and purified using QIAGEN quick gel extraction kit (QIAGEN Inc. Valencia, CA). The cDNA fragments were ligated using T4 DNA ligase (Promega, USA) as follows: (1) FG1+FG2+FG3 was ligated in an equal mole ratio and FG4+FG5(QX-S) was ligated in an equal ratio at 16°C overnight, and the appropriate size for these two ligation products was recovered from 0.8% agarose gel; (2) FG1+FG2+FG3 and FG4+FG5(QX-S) were ligated into the full-length IBV genome (Figure 1). The full-length ligation product was purified using phenol/chloroform/isoamyl alcohol (125:24:1), precipitated with 70% ethanol, and detected with electrophoresis on 0.5% agarose gel.

To produce virus genomic RNA, the full-length genomic cDNA was subjected to *in vitro* transcription using the RiboMAX^TM^ Large Scale RNA Production Systems—SP6 and T7 (Promega, USA) according to the manufacturer's instructions with modifications as described previously (Fang et al., 2007).

The N cDNA was obtained from the H120 strain and cloned into the pEASY^®^-Blunt-Zero vector. Briefly, the RNA was extracted from H120-infected allantoic fluid, and subjected to RT-PCR, to produce N cDNA with 3'-UTR. The primers for N cDNA are listed in Supplementary Table 1. The T7 promoter was incorporated at the 5' end of the N fragment, which allows the generation of N RNA by *in vitro* transcription, and a poly-A tail (30 A) was added at the 5' end, which facilitates N transcript translation. The N cDNA with T7 promoter and poly(A) tail was cloned into the pEASY^®^-Blunt-Zero vector, producing pEASY^®^-Blunt-Zero-N. The pEASY^®^-Blunt-Zero-N was digested with X*ho* I and purified. Then, the N transcript was generated using the linearized pEASY^®^-Blunt-Zero-N as a template, by using the mMESSAGE mMACHINE Ultra T7 Kit (Promega, USA). Finally, the N transcript was used for electroporation together with virus genomic RNA as a helper for the gene 1 translation and subsequent genome replication/transcription.

The full-length genomic RNA was electroporated into 5 × 10^6^ BHK-21 cells, together with the IBV N transcript. The transfected cells were cultured in DMEM (1% FBS, 100 U/ml penicillin, and 100 ug/ml streptomycin) for 48 h at 37°C, with 5% CO_2_. After 48 h, the supernatant of cell culture was collected and injected into four 10-day-old SPF chicken embryos with allantoic fluid and incubated at 37°C for 36 h. The allantoic fluid was harvested and further passed for five generations.

### Detection and Sequencing of the Rescued Virus rH120-QX(S)

To confirm the successful rescue of recombinant virus rH120-QX(S), the P5 generation of the rescued virus was checked using Western blot. Briefly, 100 μl of allantoic fluid was lysed in a 5× loading buffer (Beyotime Biotechnology, China) and denatured for 10 min at 100°C. The 10 μl samples were resolved on a 10% SDS-PAGE and transferred to a 0.45 μm nitrocellulose membrane (Pall Corporation, USA). Membranes were blocked in 5% non-fat milk for 1 h, followed by incubation with IBV N polyclonal antibody diluted in blocking buffer overnight at 4°C. The membrane was then incubated with secondary antibody diluted in blocking buffer for 1 h at room temperature. Between and after the incubation, the membrane was washed three times with washing buffer (0.1% Tween in TBS). The signals were developed with a luminol chemiluminescence reagent kit (Share-bio, China) and detected using the Tanon 4600 Chemiluminescent Imaging System (Bio Tanon, China).

To further confirm the successful rescue of rH120-QX(S), the RNA was extracted from 500 μl of allantoic fluid. RT-PCR was performed with QX S gene primers and the S gene fragment was checked by agarose gel electrophoresis. Furthermore, the full-length genome was obtained by RT-PCR (Q5^®^ super fidelity PCR kit, New England Biolabs, USA) with primers in Supplementary Table 1 and sequenced (Genewiz, China).

### Chicken Embryo Dwarf Assay

To examine the pathogenicity in chicken embryos, 100 μl of rH120-QX(S), H120, and QX strain were inoculated into the allantoic cavities of three 10-day-old SFP chicken embryos, respectively. The SFP chicken embryos were incubated for 7 days at 37°C, and the classical embryo curling was observed daily.

### ELD_50_ and EID_50_ Assay of rH120-QX(S), H120, and QX Strain

To determine ELD_50_ and EID_50_, serial 10-fold dilutions (10^−1^ to 10^−9^) of rH120-QX(S), rH120, or QX were inoculated into 10-day-old SFP chicken embryos *via* allantoic cavities (*n* = 5 each dilution). Chicken embryos that died within 1–7 days were calculated in ELD_50_. Chicken embryos with classical embryo curling were recorded in EID_50_. The ELD_50_ and EID_50_ calculations were based on the Reed and Muench method (Reed and Muench, 1938).

### Growth Curve of rH120-QX(S), rH120, and QX Strain

To compare the growth curves of the rH120-QX(S), rH120, and QX strains, 10^2^ EID_50_ of rH120-QX(S), rH120, or QX strain were inoculated into the allantoic cavities of 10-day-old SPF chicken embryos, respectively. From each embryo, 500 μl of allantoic fluid were harvested at 12, 24, 36, 48, 60, and 72 h.p.i., followed by RNA extraction and qRT-PCR. The qRT-PCR primers are IBV F391 (5'-GCTTTTGAGCCTAGCGTT-3') and IBV R533 (5'-GCCATGTTGTCACTGTCTATTG-3'). The standard curve is y = −0.282x + 11.681 (*R*^2^ = 0.997, E = 91.43%) (Callison et al., 2006). All of the assays were performed in triplicate, and the copy number of each virus was calculated based on the standard curve.

### Genome Stability

To determine the genome stability of rH120-QX(S), the virus was propagated from P1 to P20. Briefly, 100 μl of allantoic fluid containing 10^2^ EID_50_ of rH120-QX(S) was inoculated into 10-day-old SFP chicken embryos and incubated for 36 h at 37°C and was further propagated for 20 generations. The P5, P10, P15, and P20 of the virus were harvested and subjected to RT-PCR and whole-genome sequencing.

### Safety in 1-Day-Old SPF Chicken

To test the pathogenicity of rH120-QX(S), rH120, and QX strain, a total of 120 1-day-old SPF chickens were randomly assigned into four groups (A, B, C, and D), with each group containing 30 chickens being subdivided into two subgroups for clinical observation and sampling, respectively. The chickens were housed separately under negative pressure and were maintained in consistent conditions, with food and water. A, B, and C groups were infected with 10^5^ EID_50_ of rH120-QX(S), rH120, or QX strain *via* intranasal and intraocular routes, respectively. D group was inoculated with PBS as a control. Clinical signs and mortality were recorded daily until 21 days post infection (d.p.i.). On 3, 5, 7, 9, and 11 d.p.i., 2 chickens per group were randomly selected to be euthanized and subjected to necropsy. Gross tissue lesions were observed and tissue samples from the kidney, trachea, and lung were harvested. The virus load in each tissue sample was measured by qRT-PCR. On 14 d.p.i., the kidney, trachea, and lung were resected and fixed in 10% neutral formalin for 24 h and subjected to histopathological examination (HE) (Servicebio^®^, China).

### Protection Efficacy in SPF Chicken

A total of 60 1-day-old SPF chickens were randomly assigned into 3 groups (A, B, and C) of 20 chickens in each group. Groups A and B were vaccinated with 10^5^ EID_50_ of H120 or rH120-QX(S) by intranasal and intraocular routes, respectively. Group C was inoculated with sterilized PBS as a control. On 14 days post vaccination (d.p.v.), all groups were challenged with 10^4^ EID_50_ of QX *via* the intranasal and intraocular routes. All chickens were housed in negative pressure and were maintained in consistent conditions. The tissue lesion of dead chickens was examined. Meanwhile, on 3, 5, and 11 days post challenge (d.p.c.), two chickens in each group were randomly selected for dissection, examination, and detection of QX virus load. Clinical signs and mortality of chickens were recorded daily, and all chickens were examined for kidney, trachea, and lung lesions at 14 d.p.c.

### Statistical Analysis

All data are represented as the mean value ± standard derivations obtained from experiments in triplicate. All data were analyzed in GraphPad Prism version 8.0, and the statistical significance of differences between groups was evaluated using multiple *t*-tests. The significance was considered as follows: ^*^p ≤ 0.05; ^**^p ≤ 0.01; ^***^p ≤ 0.001; ^****^p ≤ 0.0001.

## Results

### Generation of rH120-QX(S) With the H120 Genomic Backbone Grafted With QX S Gene

Using RT-PCR, five fragments spanning the H120 genome, designated as FG1–FG5, were obtained from the H120 RNA genome (Figure 1A). To facilitate directional ligation of the entire genome cDNA, each fragment was flanked by B*sa* I or B*smB* I restriction enzyme recognition sequence. T7 promoter was incorporated at the 5'-terminus of FG1, which allows the generation of genomic RNA by *in vitro* transcription using T7 polymerase, and a poly-A tail (30 A) was added at the 3'-terminus of FG5, which facilitates viral protein translation (Figure 1A). The amplified PCR fragments were recovered from the agarose gel and cloned into the pEASY^®^-Blunt-Zero vector. The QX S gene (20371–23868 nt of QX genome, Genbank accession number: KY421673.1) was obtained by RT-PCR from the RNA genome, and fused with H120 FG5(ΔS) (23803–27637 nt of H120 genome), to generate the FG5(QX-S), and cloned into pEASY^®^-Blunt-Zero vector.

The five fragments were prepared by digestion from the corresponding plasmids and recovered from agarose gel, with unique cohesive overhangs (Figure 1B). The full-length cDNA was assembled *in vitro* by ligating the purified fragments at an equal mole ratio, which was in turn used as the template for *in vitro* transcription. First, FG1 (1–4,376 nt), FG2 (4,377–7,187 nt), and FG3 (7,188–14,151 nt) were ligated into a fragment covering 1–14,151 nt; FG4 (14,152–20,313 nt) and FG5(QX-S) were ligated into a chimeric fragment covering 14,152–20,313 nt of H120 genome, 20,371–23,868 nt of QX genome, and 23,803–27,637 nt of H120 genome; second, the two intermediate fragments were ligated into a full-length 27,646 nt chimeric cDNA, named rH120-QX(S) (Figure 1C).

### Rescue of the Recombinant Virus rH120-QX(S)

The full-length cDNA of rH120-QX(S) was transcribed *in vitro* using T7 polymerase. H120 N was also transcribed *in vitro* as a helper to enhance the recovery of the rescued virus (Zhou et al., 2013). The full-length genomic RNA was electroporated into BHK-21 cells, together with the N transcript. Cytopathic effects (CPE) such as cell clustering and falling-off were observed at 48 h post-transfection. To amplify the rescued virus, the supernatant of the transfected cells was collected and inoculated into allantoic cavities of 10-day-old SPF chicken embryos, and it was propagated for five generations (36 h each generation).

**Figure 2:**
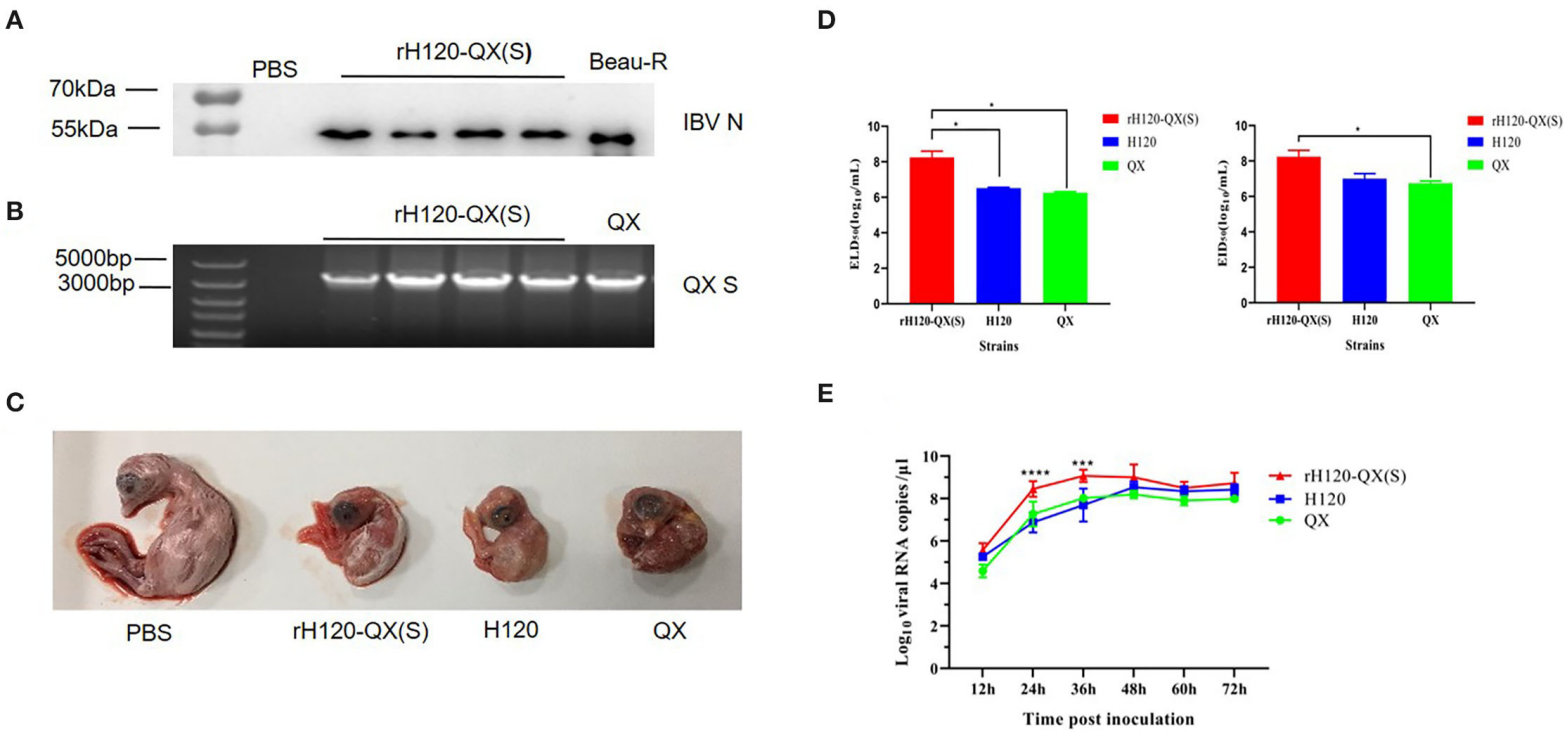
The biological characteristics of rH120-QX(S). **(A)** Western blot analysis of rH120-QX(S) in fifth-generation chicken embryos. The allantoic fluid was detected with a polyclonal antibody against the IBV N protein. The Beau-R virus was loaded as a positive control. **(B)** RT-PCR analysis of rH120-QX(S) in the fifth-generation chicken embryo. The allantoic fluid was subjected to RNA extraction and RT-PCR by using the primers targeting the QX S gene. The QX virus included in the parallel RT-PCR experiment as a positive control. **(C)** Pathogenicity of rH120-QX(S), H120, and QX strains in 10-day-old SPF chicken embryos. Embryo lesions were observed at 7 d.p.i. **(D)** The ELD_50_ and EID_50_ of the rH120-QX(S), H120, and QX strains were measured in 10-day-old SPF chicken embryos. **(E)** The growth kinetics of the rH120-QX(S), H120, and QX in 10-day-old SPF chicken embryos. *p ≤ 0.05; ***p ≤ 0.001; ****p ≤ 0.0001.

The allantoic fluid of P5 chicken embryos was collected and the recovery of the rH120-QX(S) virus was confirmed by Western Blot. As shown in Figure 2A, IBV N protein was successfully expressed in all four P5 chicken embryos, which is indicative of infectious rH120-QX(S). IBV Beau-R virus was loaded as a positive control for the detection of N protein. In addition, total RNA was extracted and analyzed with RT-PCR by using primers targeting the QX S gene. As shown in Figure 2B, a specific QX S gene was present in the recombinant virus rH120-QX(S). Sequence analysis showed no mutation in the rH120-QX(S) whole genome. Collectively, the above data demonstrate that the rH120-QX(S) was successfully rescued. The allantoic fluid was used for further passage in 10-day-old chicken embryos to generate virus stock of rH120-QX(S).

### Biological Characteristics of rH120-QX(S)

To assess the pathogenicity of rH120-QX(S) in chicken embryos, this recombinant virus was inoculated into five 10-day-old SPF embryos, and parental strain H120, S gene donor strain QX, or PBS was inoculated into parallel groups as a control. On 7 d.p.i., all the embryos inoculated with rH120-QX(S), H120, or QX strain displayed the typical IB lesions, such as dwarfing, stunted growth, curling, and death, while the PBS group did not (Figure 2C).

The ELD_50_ and EID_50_ of rH120-QX(S) were measured and calculated, by counting the dead chicken embryos or the dwarfing chicken embryos. The ELD_50_ and EID_50_ of parental strain H120 and S gene donor strain QX were also measured. As shown in Figure 2D, the ELD_50_ of rH120-QX(S) was determined as 10^8.2^ ELD_50_/ml, significantly higher than those of H120 (10^6.5^ ELD_50_/ml) and QX (10^6.3^ ELD_50_/ml) (*p* ≤ 0.05). Meanwhile, the EID_50_ of rH120-QX(S) was determined as 10^8.2^ EID_50_/ml, significantly higher than H120 (10^7^ EID_50_/ml) and QX strain (10^6.7^ EID^50^/ml) (*p* ≤ 0.05). These results indicate that rH120-QX(S) strain is more adaptable to chicken embryos than parental strain H120 and S gene donor strain QX, and it replicates in higher titers in chicken embryos.

To measure and compare the growth curve of rH120-QX(S), H120, and QX, the same dose (10^2^ EID_50_) of the three viruses was inoculated into 10-day-old SFP chicken embryos, respectively, and the allantoic fluid was harvested at 12, 24, 36, 48, 60, and 72 h.p.i. The total RNA was extracted, and the virus RNA copies were determined by qRT-PCR. Results showed that both H120 and QX strains reached the peak at 48 h with 10^8.53^ copies/μl and 10^8.19^ copies/μl, respectively, whereas rH120-QX(S) reached the peak at 36 h with 10^9.07^ copies/μl (Figure 2E). The replication level of rH120-QX(S) was higher than that of either H120 or QX.

To determine the genome stability of rH120-QX(S), the P5, P10, P15, and P20 allantoic fluids containing rH120-QX(S) were subjected to RNA extraction and RT-PCR, and the full-length cDNA was sequenced. The sequences of P5, P10, P15, and P20 of the virus were aligned with the rH120-QX(S) sequence. There was no mutation among these generations (data not shown). The above results demonstrate that the rH120-QX(S) genome is stable within 20 generations.

### Pathogenicity of rH120-QX(S), H120, and QX Strain in 1-Day-Old SPF Chicken

**Figure 3:**
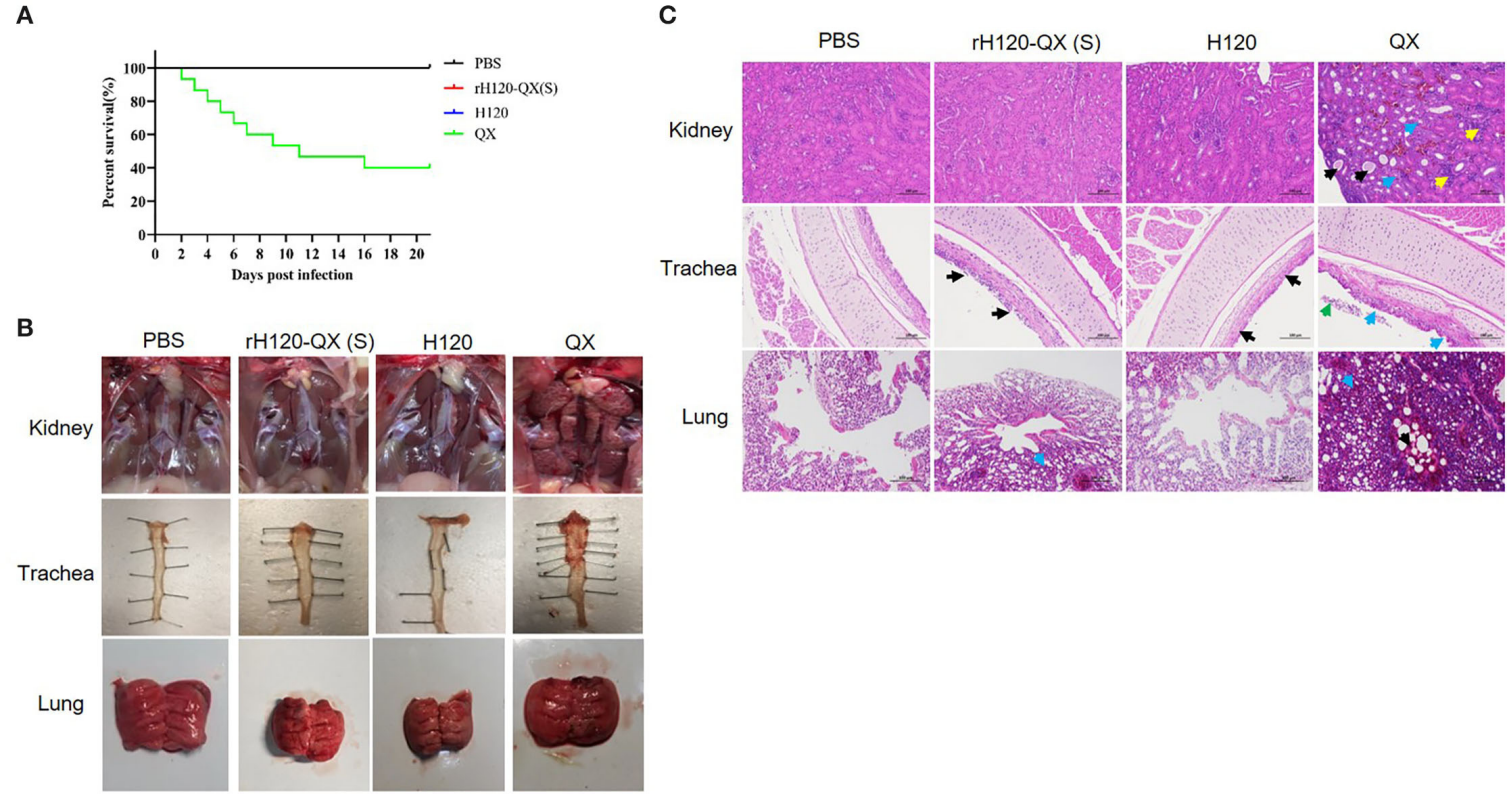
The pathogenicity of rH120-QX(S), H120, and QX strains in 1-day-old SPF chickens. **(A)** Survival percentage of 1-day-old SPF chickens infected with rH120-QX(S), H120, or QX strains. The chicken's death was observed during the period within 14 d.p.i. **(B)** Representative gross lesions on the trachea, kidney, and lung 14 d.p.i. **(C)** Histopathological changes of the trachea, kidney, and lung on 14 d.p.i.

To evaluate the safety of rH120-QX(S), 30 1-day-old SPF chickens of each group were infected with 10^5^ EID_50_ of rH120-QX(S), H120, or QX strain, by eye-drop and intranasal inoculation. PBS was inoculated in a parallel-group as a negative control. Clinical signs were observed and recorded up to 14 d.p.i., including snicking, wheezing, and nasal discharge. Results showed that H120 or rH120-QX(S) infection caused a slight hemorrhage in the trachea, no visible lesions were observed in the kidney and lung, and all chickens survived up to 14 h.p.i. However, QX strain exhibited high pathogenicity to 1-day-old SPF chickens, with typical clinical signs such as depression and rale. In the QX-infected group, the earliest death was observed at 2 d.p.i., and the mortality rate was up to 60% (Figure 3A). Necropsy showed there was swelling and urate deposition in the kidney, and there was severe hemorrhage, mucus, hyperemia, edema, and congestion in the lung, whereas H120, rH120-QX(S), or PBS-treated group did not show these tissue damages (Figure 3B). These results suggest that the replacement of the H120 S gene with the QX S gene does not confer pathogenicity to the rH120-QX(S) virus.

Subsequently, the kidney, trachea, and lung of four groups were resected for histopathological examination (HE) on 14 d.p.i. (Figure 3C). The HE staining results were consistent with the gross lesions in Figure 3B. Similar to the PBS-treated group, no kidney lesions were observed in the rH120-QX(S)- or H120-infected group; however, obvious kidney lesions were observed in the QX-infected group, which were characterized as tubular dilatation (indicated with black arrows), necrosis in the epithelial cells of renal tubules (indicated with blue arrows), and abundant erythrocyte/lymphocyte infiltration (indicated with yellow arrows). In the trachea, the local irregular cilia were observed in the H120-infected group (indicated with black arrows); chickens inoculated with rH120-QX(S) presented with moderate cilia loss (indicated with black arrows); whereas cilia-shedding in the epithelium (indicated with blue arrows) and necrosis in the lumen (indicated with green arrows) were observed in the QX-infected group. Lesions in the lung consisted of congestion and hemorrhage (indicated with black arrows), as well as capillary stenosis (indicated with blue arrows) in the QX-infected group; in contrast, there was no lesion observed in the lung of the H120-infected chicken, and mild capillary congestion was observed in the rH120-QX(S)-infected chicken; no lesions were observed in the PBS-treated group. These results support that the virulence of the rH120-QX(S) strain is attenuated and is safe for chickens.

### Tissue Tropisms of the rH120-QX(S)

**Figure 4:**
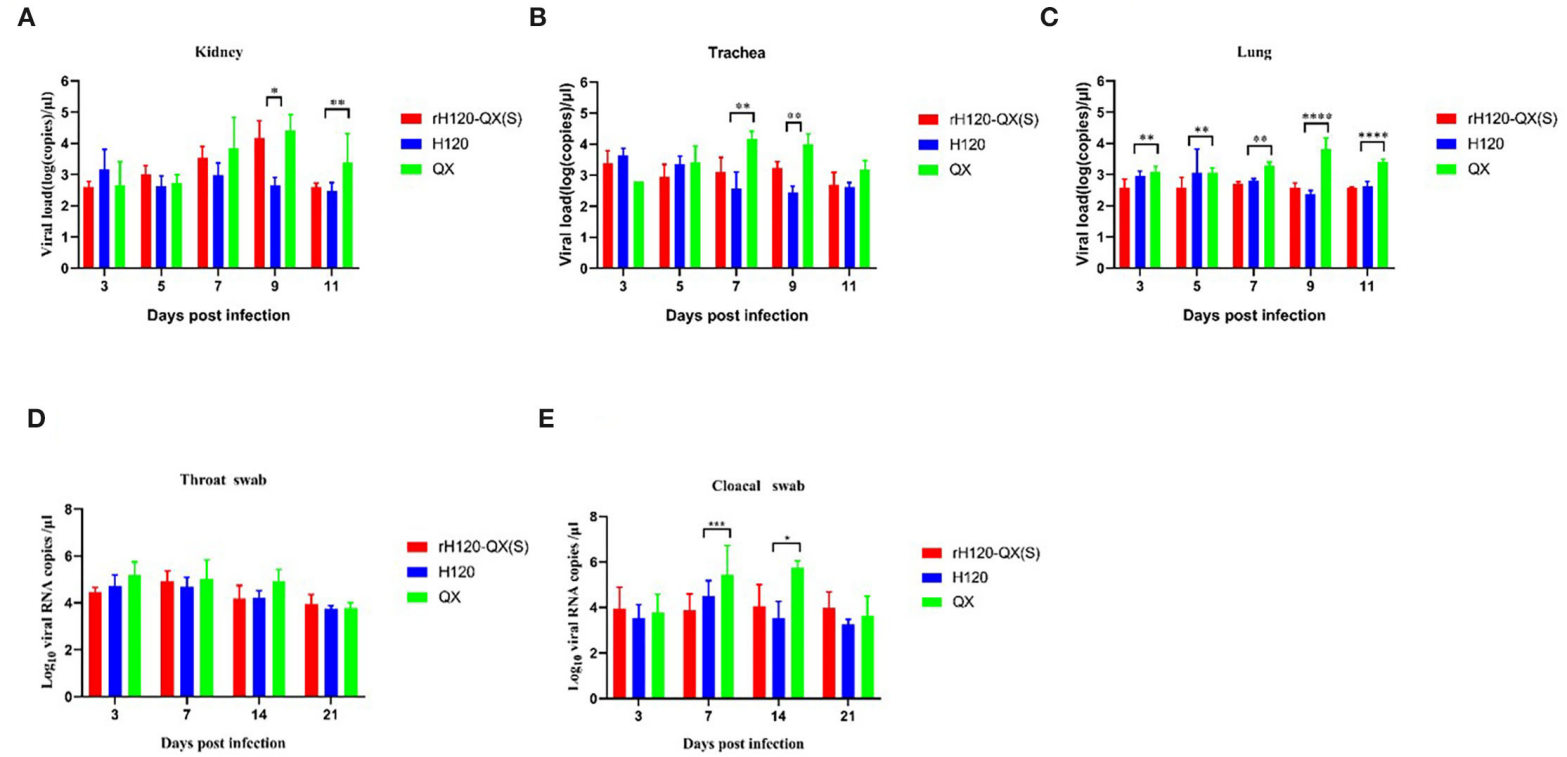
Virus load in various tissues in SPF chickens infected with rH120-QX(S), H120, or QX. Virus load in the kidney **(A)**, trachea **(B)**, lung **(C)**, and was measured by RT-PCR on 3, 5, 7, 9, and 11 d.p.i. **(D,E)** Virus load in the throat and cloacal of the SPF chicken infected with rH120-QX(S), H120, or QX. Virus load was measured by RT-PCR on 3, 7, 14, and 21 d.p.i. *p ≤ 0.05; **p ≤ 0.01; ***p ≤ 0.001; ****p ≤ 0.0001.

To investigate the tissue tropisms of the rH120-QX(S), one-day-old SPF chickens were infected with H120, rH120-QX(S), or QX strain, respectively. A range of tissues (kidney, trachea, and lung) were collected on 3, 5, 7, 9, and 11 d.p.i., and the virus load was assessed by qRT-PCR. As shown in Figures 4A–C, the virus load of QX in all tissues was gradually increased and peaked at 7–9 d.p.i., which were higher than the rH120-QX(S) or H120 infection group, indicating the highest replication level of this field virus strain. The load of rH120-QX(S) was comparable to that of H120, except for in the kidney and trachea on 9 d.p.i. These results demonstrate that the recombinant strain rH120-QX(S) displays similar tissue tropisms and replication level to the vaccine strain H120.

To determine the virus excretion, the virus load in the throat and cloacal was measured with qRT-PCR on 3, 7, 14, and 21 d.p.i. As shown in Figures 4D,E, on 7 and 14 d.p.i., the QX load in the cloacal swab is significantly higher than rH120-QX(S) and H120; however, there was no significant difference between rH120-QX(S), H120, and QX in the throat. All the three viruses were declined in both the throat and cloacae on 21 d.p.i.

### Protective Efficacy of rH120-QX(S)

**Figure 5:**
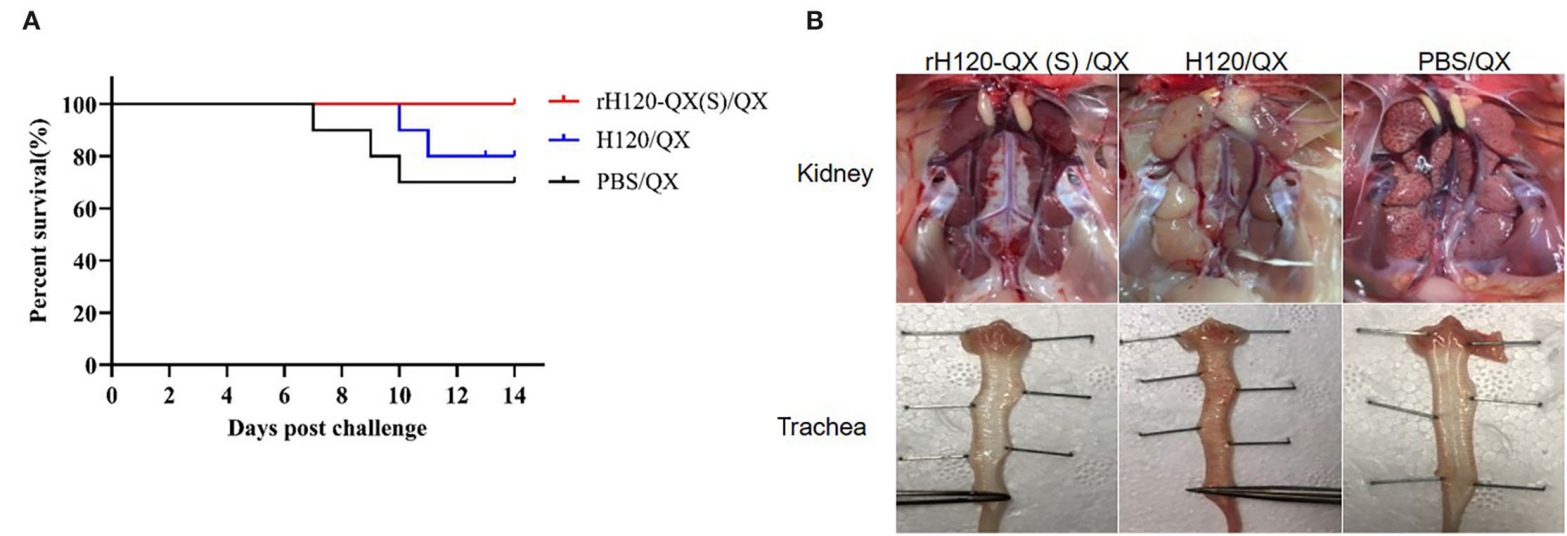
Protection efficacy of rH120-QX(S). 1-day-old SPF chickens were vaccinated with H120, rH120-QX(S), or treated with PBS, followed by QX challenge. **(A)** The chicken death was observed during the period up to 14 d.p.c., and the survival rate was calculated. **(B)** Gross lesions were observed in the kidney and trachea.

To examine whether rH120-QX(S) protects the pathogenic QX strain, 20 1-day-old SPF chickens in each group were immunized with 10^5^ EID_50_ of rH120-QX(S) or H120, respectively. PBS was inoculated in a parallel-group as a control. On 14 d.p.v., chickens were challenged with 10^4^ EID_50_ of QX, followed by observation for 14 days. Results showed that all the chickens immunized with rH120-QX(S) survived without clinical signs after being challenged with QX (Figure 5A). After necropsy, lesions in the kidney and respiratory tract were examined. There were no tissue lesions observed in the kidney and trachea (Figure 5B). However, in chickens immunized with H120 or treated with PBS, there were typical clinical signs after challenge with QX, such as mouth breathing and rale, and the mortality rate reached 20% (immunized with H120) and 30% (immunized with PBS), respectively (Figure 5A). In the dead chickens of the PBS-immunized group, the kidney showed typical “flower-spotted kidney” due to swelling and white urate deposits, and the throat exhibited slight hemorrhage; in the H120-immunized group, the dead chicken kidney showed swollen and pale discoloration, and the trachea displayed severe extensive hemorrhage (Figure 5B). These results showed that rH120-QX(S) confers 100% protection to the QX strain, while H120 provides limited protection.

**Figure 6:**
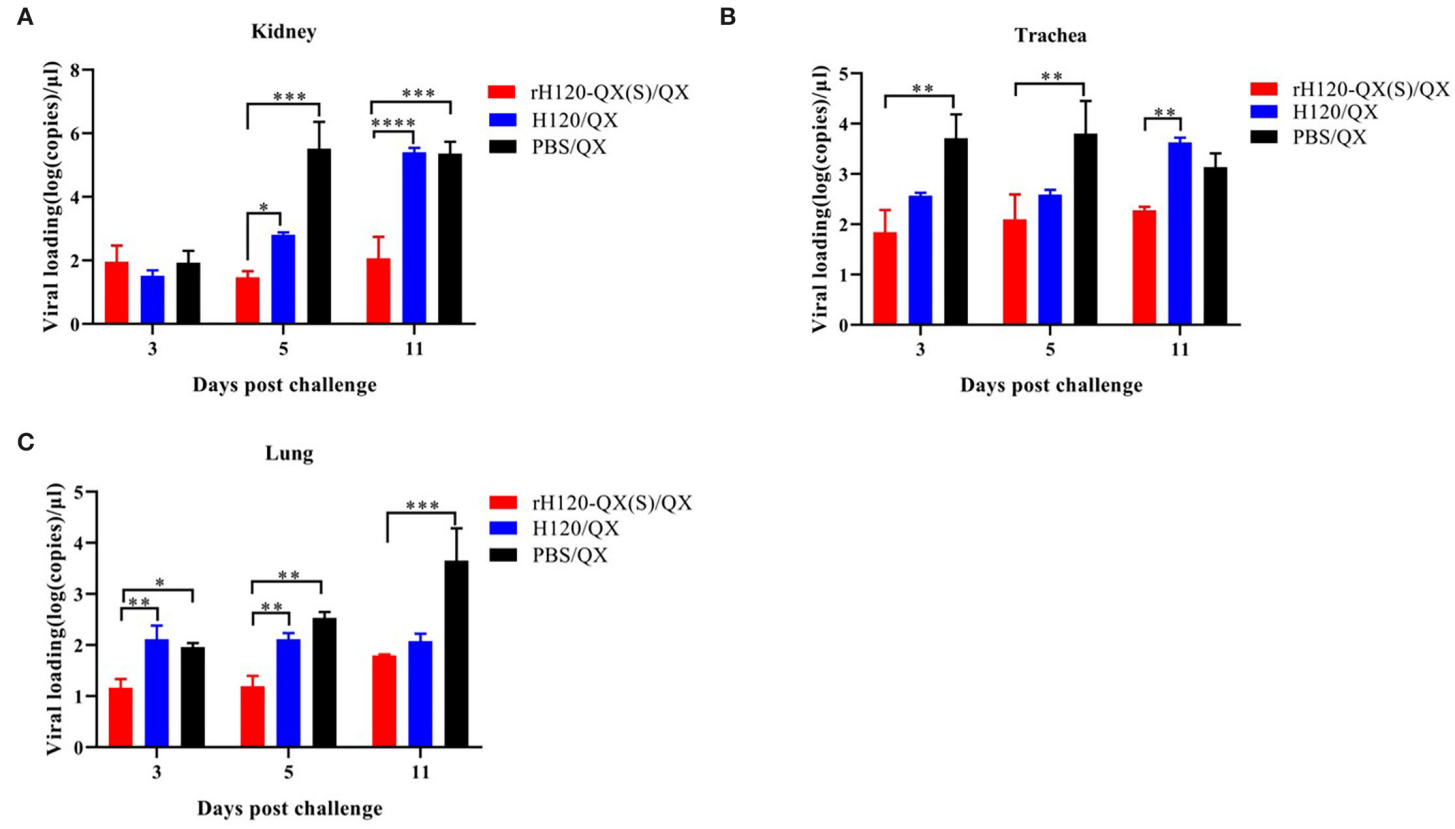
QX virus load in the kidney, trachea, or lung of SPF chickens vaccinated with rH120-QX(S), H120, or treated with PBS, followed by QX challenge. **(A)** QX virus load in the kidney on 3, 5, and 11 d.p.c. **(B)** QX virus load in the trachea on 3, 5, and 11 d.p.c. **(C)** QX virus load in the lung on 3, 5, and 11 d.p.c. *p ≤ 0.05; **p ≤ 0.01; ***p ≤ 0.001; ****p ≤ 0.0001.

To check the QX virus load in the chicken vaccinated with rH120-QX(S) or H120 and subsequent QX challenge, the kidney, trachea, and lung were collected on 3, 5, and 11 d.p.i., and the virus load was assessed with qRT-PCR. As shown in Figure 6, in all three tissues, the QX virus load in rH120-QX(S)-vaccinated chickens (rH120-QX(S)/QX) was significantly lower than those of PBS-treated or H120-vaccinated chickens (PBS/QX or H120/QX), except for the virus load in the kidney on 3 d.p.c.; PBS-treated group (PBS/QX) displayed the highest QX virus load. These results demonstrate that rH120-QX(S) provides stronger protection against QX than the H120 vaccine.

## Discussion

Vaccination with live-attenuated vaccines is the most effective strategy to control the IB epidemic in poultry farms (Britton et al., 2012; Yun et al., 2021). However, the currently available IBV vaccines provide limited cross-protection to different serotypes/genotypes, and new variant strains frequently emerge, making the elimination and control of IB difficult (Wit et al., 2011; Bande et al., 2017). In recent years, increasing cases of IBV outbreaks have been detected in poultry farms, and QX-type IBV remains the prevalent genotype in many countries (Zou et al., 2010; Khataby et al., 2016; Ismail et al., 2020). Consequently, it is necessary to generate IBV vaccines against the newly emerging field strains using a precise and quick technique to modify the virus genome and attenuate the virus virulence. A reverse genetics technique for the IBV Beaudette strain has been established since 2001 (Casais et al., 2001). The maturation of this technique makes it possible to develop vaccines by targeted mutation or specific recombination (Zhou et al., 2013). In this study, we successfully constructed the infectious clone of traditional IBV vaccine strain H120 by the *in vitro* ligation method and grafted the S gene of prevalent strain QX onto the backbone of the H120 to generate a recombinant vaccine candidate with protection against virulent QX.

The IBV S protein, a type I membrane glycoprotein with S1 and S2 subunits, mediates virus entry and harbors the antigens for producing specific neutralization antibodies. Thus, this protein is a suitable protective antigen for vaccine development. Along with the successful development of reverse genetics of Beau-R, the Vero cell adaptive strain, several reports attempt to produce the recombinant virus by grafting the prevalent IBV strain S protein to avirulent Beau-R. The 4/91 S gene was successfully grafted onto the Beau-R genome to produce BeauR-4/91(S) with the Beau-R backbone and 4/91 S gene (Armesto et al., 2011). Vaccination with BeauR-4/91(S) and challenging with 4/91 showed the absence of clinical symptoms and induction of protective immunity against the wild-type 4/91 (Armesto et al., 2011). Similarly, a recombinant virus BeauR-M41(S) remained apathogenic but conferred protection against M41 (Hodgson et al., 2004). There is evidence that two recombinant viruses expressing M41 strain S1 or S2 based on the Beau-R backbone did not protect as effectively as BeauR-M41(S) (Ellis et al., 2018), suggesting that the full-length S protein is necessary for full protection. However, there is one exception: IBYZ strain S1 subunit, rather than the full-length S gene, still induces high humoral antibody titers to provide effective protective immunity against IBYZ when chimeric into H120 (Jiang et al., 2020). Thus, further investigation was required to clarify whether grafting of the S1 subunit or RBD is enough for neutralization protection. The Beau-R strain is not a vaccine strain and is only used in the lab because of its cell culture adaptation characteristics. The introduction of the Beau-R backbone into recombinant vaccines will raise the risk of increasing the recombinant rate among the vaccines (such as H120, H52, and Beau-R backbone) or among the recombinant vaccines and field strains. Considering biosafety issues of increasing the risk of generating new recombinant viruses, we should be careful to introduce the Beau-R strain into poultry farms, although the Beau-R strain is apathogenic. Traditional vaccine strain is more suitable as the backbone for the development of recombinant vaccines. H120 is the earliest and representative attenuated live IBV vaccine against the Mass genotype. This vaccine has been used on poultry farms worldwide for decades. Here, we chose the H120 as the recombinant vaccine backbone, not only for safety but also for reducing recombination risk.

In this study, we developed a high-efficiency reverse genetics system of H120, by *in vitro* ligation of five contiguous fragments of size 3–7 kb to produce genomic full-length cDNA. This strategy is convenient to ligate full-length cDNA by decreasing the number of cDNA fragments, similar to the strategy of SARS-CoV-2 (Xie et al., 2020). Based on this H120 recombinant platform, we constructed a recombinant virus rH120-QX(S) by grafting the field virulent strain QX S gene. This recombinant virus is more adaptive to chicken embryos, with a higher ELD_50_ and EID_50_ than the parental H120 or donor QX strain. Although rH120-QX(S) still exhibits pathogenicity in chicken embryos, it is nonpathogenic in SPF chicken, comparable to the parental H120 strain, whereas the lethality of the donor strain QX is up to 60%, similar to the previously described (Shao et al., 2020). Thus, the QX S protein does not confer virulence to rH120-QX(S). In the protection experiment, we found that the rH120-QX(S) strain confers better and more effective protection against QX than the H120 vaccine.

In all, this study provides a key tool for precisely and rapidly generating live-attenuated IBV vaccine candidates by reverse genetics. The *in vitro* ligation method in this system offers an effective way to design highly protective and inexpensive recombinant vaccines. The rH120-QX(S) produced by this method is a good vaccine candidate for controlling the prevalent strain QX.

## Data Availability Statement

The datasets presented in this study can be found in online repositories. The GenBank accession numbers for nucleotide sequences of H120 and rH120-QX(S) are ON350836 and ON350837.

## Ethics Statement

The animal study was reviewed and approved by the Animal Welfare and Ethical Censor Committee of Shanghai Veterinary Research Institute, Chinese Academy of Agricultural Sciences.

## Author Contributions

YL designed and conceptualized this project. WW, QL, and HW performed the experiments. SF provided the technical support for the reverse genetics of IBV. YS, LT, CS, XQ, and WL provided the resources and supervised the animal experiments. WW and WX wrote the original draft. YL reviewed the data and edited the manuscript. YL and CD contributed to funding acquisition and supervision. All authors read and approved the final manuscript.

## Funding

This study was supported by the National Natural Science Foundation of China (32172834) and the National Key Research and Development Program (No. 2021YFD1801104).

## Conflict of Interest

The authors declare that the research was conducted in the absence of any commercial or financial relationships that could be construed as a potential conflict of interest.

## Publisher's Note

All claims expressed in this article are solely those of the authors and do not necessarily represent those of their affiliated organizations, or those of the publisher, the editors and the reviewers. Any product that may be evaluated in this article, or claim that may be made by its manufacturer, is not guaranteed or endorsed by the publisher.

## Supplementary Material

Supplementary Table 1 Primers for amplifying the fragments of genome cDNA of H120, QX S gene, and H120 N gene.
