# Supplementary material for: Characterization of the Protective Efficacy Against QX Strain of a Recombinant Infectious Bronchitis Virus With H120 Backbone and QX Spike Gene
Source: Front Microbiol. 2022 Jun 17;13:883642. doi: 10.3389/fmicb.2022.883642 (PMC9247577; doi:10.3389/fmicb.2022.883642)
Supplement: Supplementary Table 1 — Primers for amplifying the fragments of genome cDNA of H120, QX S gene, and H120 N gene. [file Table_1.DOCX]

**Table 1** Primers for amplifying the fragments of genome cDNA of H120, QX S gene, and H120 N gene.

| **Primers** | **Sequences (5'-3')** |
| --- | --- |
| FG1-F | GC***GAGACG***TAATACGACTCACTATAGGGACTTAAGATAGATATTAATATATATCTATTGCA (B*sm*B I, 1-33 nt) |
| FG1-R | T***GCGTCT***CCACCCAAAAGTT (B*sm*B I, 4,370-4,386 nt) |
| FG2-F | ACAACTTTTGGGTG***GAGACC***CAACT (B*sa* I, 4,368-4,392 nt) |
| FG2-R | CCAGATCTTGTTATAAAGAAAC***GAGACC*** (B*sa* I, 7,181-7,208 nt) |
| FG3-F | ***GGTCTC***GTTTCTTTATAACAAGATCTG (B*sa* I, 7,181-7,207 nt) |
| FG3-R | ***GGTCTC***TGCCCTATCACACTTTGG (B*sa* I, 14,139-14,156 nt) |
| FG4-F | ***GGTCTC***AGGGCAATGCCTAATTTGT (B*sa* I, 14,151-14,169 nt) |
| FG4-R | ***GGTCTC***AACATCTCTTACCAGTAACTTAC (B*sa* I, 20,296-20,319) |
| FG5(△S)-F | CCTAAAAAGTCTGTTTAATGATCCAAAGTCCCACGTCCTTCTTAATAG (23,803-23,832 nt) |
| FG5(△S)-R | ***GGTCTC***GAGTTTTTTTTTTTTTTTTTTTTTTTTTTTTTTTGCTCTAACTCTATACTAGCCTATAA (B*sa* I, 27,582-27,637 nt) |
| QX-S-F | ***GGTCTC***GATGTTGGGGAAGTCACTG (B*sa* I, 20,370-20,388 nt) |
| QX-S-R | GACGTGGGACTTTGGATCATTAAACAGACTTTTTAGGTCTGTATTGTTC (23,839-23,868 nt) |
| N-F | TAATACGACTCACTATAGGGATGGCGAGCGGTAAGACAACTG (25,873-25,894 nt) |
| N-R | ***CTCGAG***TTTTTTTTTTTTTTTTTTTTTTTTTTTTTTTGCTCTAACTCTATACTAGCCTATAA（X*ho* I, 27,582-27,637 nt） |
